# Association of age and cause-special mortality in patients with stage I/ II colon cancer: A population-based competing risk analysis

**DOI:** 10.1371/journal.pone.0240715

**Published:** 2020-10-16

**Authors:** Huajun Cai, Yiyi Zhang, Xing Liu, Weizhong Jiang, Zhifen Chen, Shoufeng Li, Guoxian Guan

**Affiliations:** 1 Department of Colorectal Surgery, The First Affiliated Hospital of Fujian Medical University, Fuzhou, China; 2 Department of Colorectal Surgery, Fujian Medical University Union Hospital, Fuzhou, China; Chang Gung Memorial Hospital at Linkou, TAIWAN

## Abstract

**Purpose:**

This study aimed to determine the probability and prognostic factors of colon cancer-specific mortality (CCSM) and noncancer-specific mortality (NCSM) for patients with stage I/II colon cancer and evaluate the association of age on cause-specific mortality.

**Materials and methods:**

From Surveillance, Epidemiology, and End Results (SEER) database, we identified 33152 patients with stage I/II colon cancer undergoing surgery between 2004 and 2011. The cumulative incidence of CCSM and NCSM was calculated, and competing risk analysis was performed to investigate prognostic factors for cause-specific mortality.

**Results:**

In patients <50, 50–75, and >75 years of age, 5-year cumulative incidence of CCSM was 5.7%, 7.8%, and 16.1%, respectively (overall, 10.6%); 5-year cumulative incidence of NCSM was 2.2%, 7.1%, and 26.9%, respectively (overall, 13.8%). The probability of CCSM and NCSM increased with advanced age. The 5-year cumulative incidence of CCSM was higher than NCSM in patients <50 years of age, whereas lower in patients >75 years of age. The probability of CCSM and NCSM was similar in patients 50–75 years of age. Competing-risk multivariable analysis demonstrated that increasing age was a strong predictor of CCSM (per year increase, SHR 1.03,95% confidence interval [CI]: 1.03–1.04). Age was most predictive of NCSM: (per year increase, SHR 1.08, 95% CI: 1.08–1.08).

**Conclusion:**

Age was significantly associated with an increased cumulative incidence of CCSM and NCSM of patients with stage I/II colon cancer underwent surgery. NCSM was a significant competing event and should be adequately considered when performing survival analysis.

## Introduction

Colon cancer is one of the most common malignancies worldwide. Due to the development of diagnosis and treatment, the oncology prognosis of colon cancer has improved [1]. However, during long-term follow-up, noncancer-specific death, such as cerebrovascular diseases and diseases of heart, so-called competing event, is one of the main causes of death, especially for cancers with long survival time. Considering the good prognosis of stage I/II colon cancer, the benefit of treatment should take competing risk of death into consider. On another hand, over 30% of diagnosed patients with colon cancer are 75 years or older, this proportion may also increase due to the aging population [2]. In oncology research, some elderly patients will die from competing event before reaching the end point of interest. Given ageing comes with comorbid diseases and frailty, the risk of competing events increases with age [3, 4]. Therefore, competing risk should be taken into account when evaluating the prognosis.

Previous studies have reported the relationship between age and colon cancer prognosis [5–8]. However, most studies limited to the analysis of overall survival or cancer-specific mortality or used conventional Kaplan-Meier survival analysis and Cox regression models analyzed cancer-specific mortality. In the era of individualized cancer therapy, both cancer and noncancer risk factors should be taken into consideration when tailoring treatments. When there are competing events in survival data analysis, Kaplan-Meier method and Cox regression models may be inappropriate because they treat competing events as independent censoring, which will lead to bias in survival data analysis [9–11]. In this case, competing risks analysis is recommended.

Therefore, we used data from the Surveillance, Epidemiology, and End Results (SEER) program to compare clinicopathological characteristics and survival outcome of patients with stage I/II colon cancer. We further performed a competing risks analysis to evaluate the prognostic impact of age on cause-specific mortality in order to guide postoperative therapy and surveillance programme.

## Material and methods

### Ethics statement

The study was approved by the Institutional Review Board of the First Affiliated Hospital of Fujian Medical University. Because cases were collected from the SEER database and were analyzed anonymously, therefore no additional informed consent was required.

### Data source

The National Cancer Institute’s SEER project consists of 18 population-based cancer registries that cover approximately 28% of the US population [12]. The SEER project collects data which include cancer demographics, clinicopathological features, and survival data. SEER data do not contain identifiers and can be published for research on cancer-based epidemiology and survival analysis. The SEER-stat software (the Surveillance Research Program, National Cancer Institute SEER Stat software, www.seer.cancer.gov/seerstat, Version 8.3.5) was used to identify the study cohort. This study was based on public data with the reference number 13886-Nov2017. It did not involve human subjects or personal information, and informed consent was not required.

### Study cohort

**Fig 1:**
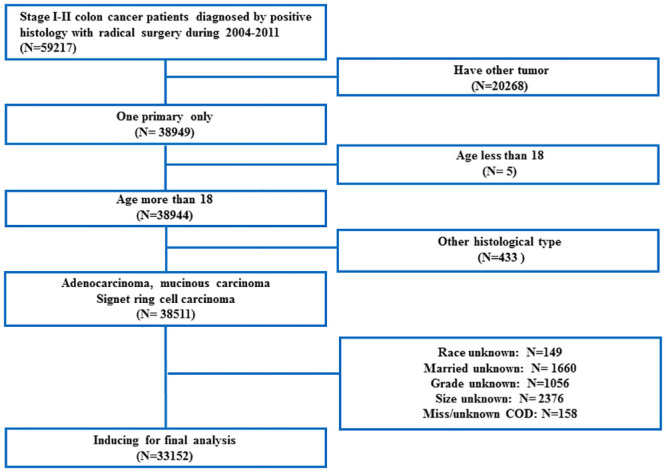
Flow chart of patient cohort definitions. COD: cause of death.

We used the SEER-stat software to identify colon cancer patients who underwent radical surgery between 2004 and 2011. Patient inclusion criteria were: 1) pathological stage I/II disease, 2) aged >18 years and 3) pathologically confirmed adenocarcinoma, mucinous carcinoma, or signet ring cell carcinoma. Exclusion criteria included 1) concurrent with another tumor during lifetime, 2) other histological types, and 3) unknown information about race, marriage, grade, and size. The flow chart of patient enrollment was shown in Fig 1.

Demographic and clinicopathological data were extracted from the SEER database, including age, sex, race, marital status, tumor location, tumor size, histological type(SEER histology codes 8140, 8210–11, 8220–21, 8260–63, 8480–81, 8490), histological grade, T stage, follow-up data, and cause of death. Patients were stratified into three groups according to age at diagnosis (<50, 50–75, and >75 years) when calculated the cumulative incidence of CCSM and NCSM. Tumor location was distributed according to the primary site: the right colon (C18.0, C18.2-C18.4) and the left colon (C18.5-C18.7). Tumor size was converted from a continuous variable to a categorical variable with a cut-off value of 5.0 cm. TNM classification was restaged according to the criteria described in the American Joint Committee on Cancer (AJCC) Cancer Staging Manual (7th edition, 2010). The endpoints of the study were cause-specific mortality, including colon cancer-specific mortality (CCSM) and noncancer-specific mortality (NCSM). CCSM was defined as death as a result of recurrent disease following radical resection. NCSM was defined as death due to a specific reason rather than a malignant tumor. CCSM and NCSM were considered to be two competing events. Cause of death investigated by SEER cause-specific death classification and SEER other cause of death classification in SEER database. Exactly, SEER cause-specific death classification designates that the person died of their cancer for cause-specific survival, including alive or dead of other cause, dead (attributable to this cancer) and dead (missing/unknown cause of death). SEER other cause of death classification designates that the person died of causes other than their cancer, including alive or dead due to cancer, dead (attributable to causes other than this cancer) and dead (missing/unknown cause of death). Our study excluded patients with unknown cause of death, therefore we can measure the cause of death for cancer-specific or not.

### Statistical analysis

All analyses were performed using SPSS software version 24.0 (IBM SPSS INC., Chicago, USA) and R software version 3.5.1 (The R Foundation for Statistical Computing, Vienna, Austria; www.rproject.org). Clinicopathological characteristics were described by use of proportions. Standardized differences were used to estimate whether these demographic and clinicopathological features were balanced. Standardized differences of less than 10 per cent have been found to reflect well balanced demographic and clinicopathological features [13]. The 5-year cumulative incidence of death (CID) was estimated by using the cumulative incidence function and the difference between age groups was estimated by the Gray test [14]. Multivariable analyses using the Fine and Gray model were performed to identify prognostic factors for CCSM and NCSM [15]. We also used restricted cubic splines to estimate the age-varying association of cause-special mortality by competing risk model. The subdistribution hazard curve against 75 years was plotted smoothly. Kaplan-Meier method and cause-specific hazard models were also performed to provide complimentary information. Our study included patients with unknown demographic and clinicopathological data into statistical analysis as supplementary data to exclude the influence of unknown information. Sub-distribution analysis, cause-specific hazard models and survival analysis of competitive risk were calculated by using R package cmprsk and survival. All statistical tests were two-sided, and *P*<0.05 was considered statistically significant.

## Results

### Patient characteristics

A total of 33152 patients were included in this study, including 152324 (46.2%) males and 17828 (53.8%) females. Among them, 30174 (91.1%) patients were >50 years of age, including 11890 (35.9%) >75 years old. The median follow-up time was 71 (interquartile range: 50–97) months. The baseline characteristics of the patients are summarized in Table 1. A significantly increased proportion of female and right colon patients were observed as the age increased. Patients <50 years of age tended to have more large tumors.

**Table 1:** Characteristics of colon cancer patients by age-group.

Characteristic	Total (%) (n = 33152)	Age group, number (%)			Standardized difference (%)*		
		<50 years (n = 2978)	50–75 years (n = 18284)	>75 years (n = 11890)	<50 years *versus* 50–75 years	<50 years *versus* >75 years	50–75 years *versus* >75 years
**Sex**							
Male	15324 (46.2)	1535 (51.5)	9411 (51.5)	4378 (36.8)	0	-14.7	-14.7
Female	17828 (53.8)	1443 (48.5)	8873 (48.5)	7512 (63.2)	0	14.7	14.7
**Race**							
White	26969 (81.3)	2215 (74.4)	14308 (78.3)	10466 (88.0)	3.9	13.5	9.6
Non-white	6183 (18.7)	763 (25.6)	3976 (21.7)	1444 (12.0)	-3.9	-13.5	-9.6
**Marital status**							
Married	18531 (55.9)	1843 (61.9)	11549 (63.2)	5139 (43.2)	1.3	-18.7	-19.9
Unmarried	14621 (44.1)	1135 (38.1)	6735 (36.8)	6751 (56.8)	-1.3	18.7	19.9
**Tumor location**							
Right-colon	22501 (67.9)	1589 (53.4)	11828 (64.7)	9084 (76.4)	11.3	24.0	11.7
Left-colon	10651 (32.1)	1389 (46.6)	6456 (35.3)	2806 (23.6)	-11.3	-24.0	-11.7
**Tumor size, cm**							
<5	19232 (58.0)	1340 (45.0)	11064 (60.5)	6828 (57.4)	15.5	12.4	-3.1
≥5	13920 (42.0)	1638 (55.0)	7220 (39.5)	5062 (42.6)	-15.5	-12.4	3.1
**Histological type**							
Adenocarcinoma	29596 (89.3)	2637 (88.5)	16518 (90.3)	10441 (87.8)	1.8	-0.7	-2.5
MC/SRCC	3556 (10.7)	341 (11.5)	1766 (9.7)	1449 (12.2)	-1.8	0.7	2.5
**Histological grade**							
Well/Moderately	28123 (84.8)	2539 (85.3)	15858 (86.7)	9726 (81.8)	1.5	-3.5	-4.9
Poorly/Undifferentiated	5029 (15.2)	439 (14.7)	2426 (13.3)	2164 (18.2)	-1.5	3.5	4.9
**T stage**							
T1-2	10986 (33.1)	799 (26.8)	6491 (35.5)	3696 (31.1)	8.7	4.3	-4.4
T3	19279 (58.2)	1858 (62.4)	10266 (56.1)	7155 (60.2)	-6.2	-2.2	4.0
T4	2887 (8.7)	321 (10.8)	1527 (8.4)	1039 (8.7)	-2.4	-2.0	0.4

MC: mucinous carcinoma; SRCC: signet-ring cell carcinoma

*Standardized differences with absolute values of less than 10 per cent reflect well balanced co-variables [13]

### Cause-specific mortality by age group

**Fig 2:**
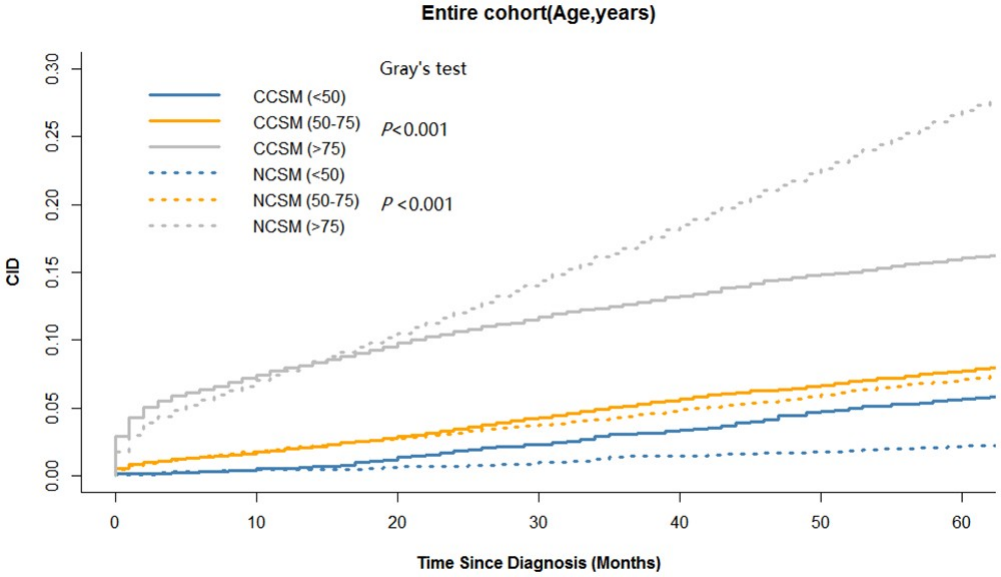
Colon cancer–specific and noncancer-specific 5-year cumulative incidence of death by age-group. CCSM: colon cancer-specific mortality; NCSM: noncancer-specific mortality; CID: cumulative incidence of death.

**Fig 3:**
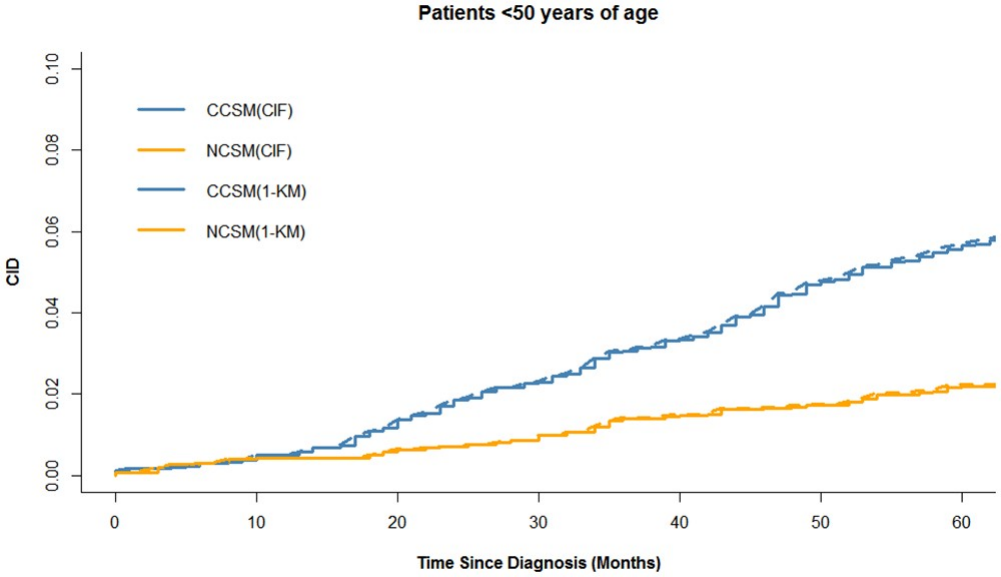
Cumulative incidence functions and Kaplan-Meier method estimates for patient <50 years of age. CCSM: colon cancer-specific mortality; NCSM: noncancer-specific mortality; CID: cumulative incidence of death; CIF: Cumulative incidence functions; KM: Kaplan-Meier method.

**Fig 4:**
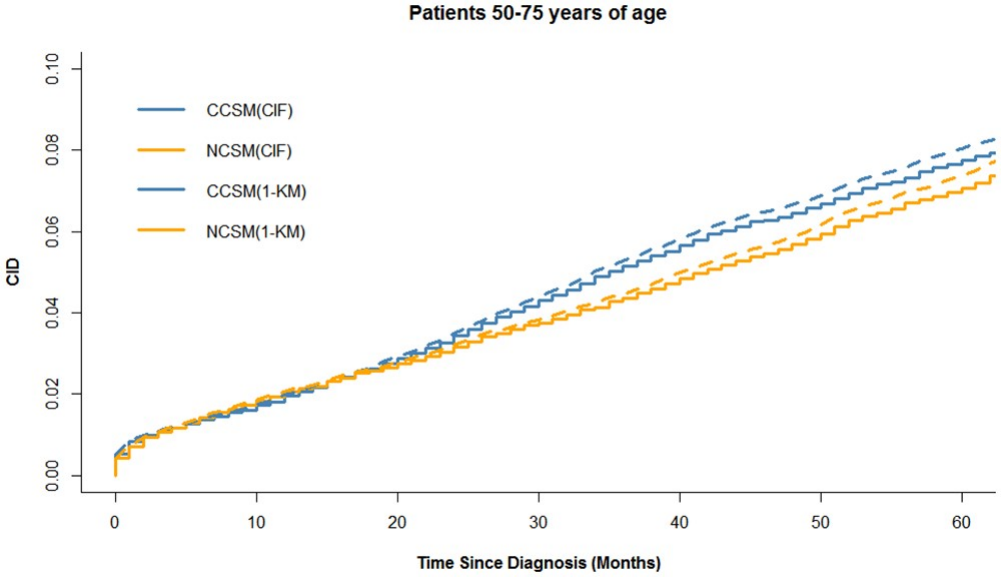
Cumulative incidence functions and Kaplan-Meier method estimates for patient 50–75 years of age. CCSM: colon cancer-specific mortality; NCSM: noncancer-specific mortality; CID: cumulative incidence of death; CIF: Cumulative incidence functions; KM: Kaplan-Meier method.

**Fig 5:**
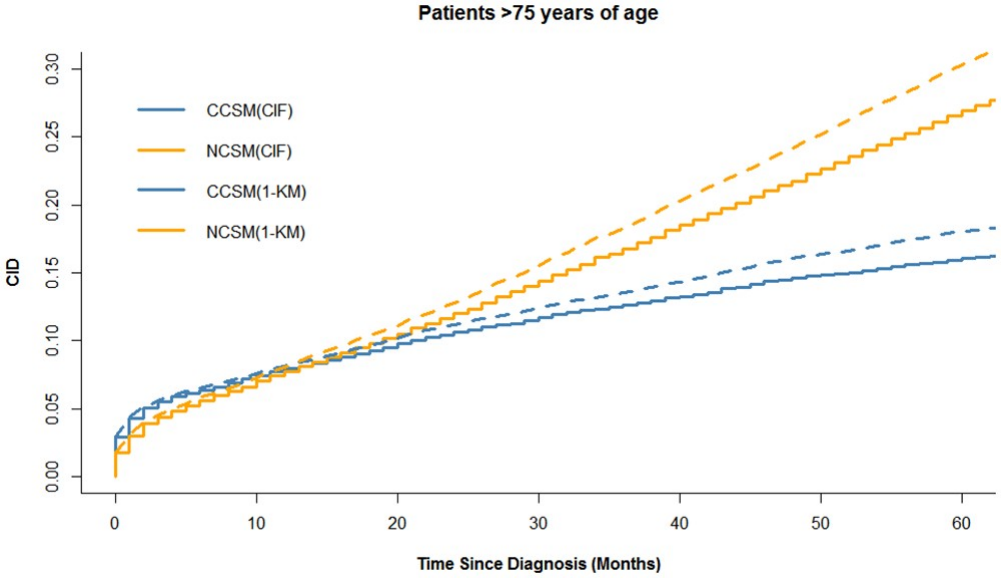
Cumulative incidence functions and Kaplan-Meier method estimates for patient >75 years of age. CCSM: colon cancer-specific mortality; NCSM: noncancer-specific mortality; CID: cumulative incidence of death; CIF: Cumulative incidence functions; KM: Kaplan-Meier method.

The colon cancer-specific and noncancer-specific CID curves are shown in Fig 2. In the whole cohort, the 5-year colon cancer-specific and noncancer-specific CID were 10.6% (95%CI:10.2–10.9%) and 13.8% (95%CI: 13.4–14.2%), respectively. Among patients <50, 50–75, and >75 years of age, the 5-year colon cancer-specific-CID were 5.7% (95%CI:4.8–6.5%), 7.8% (95%CI:7.4–8.1%), and 16.1% (95%CI: 15.4–16.7%), and the difference of the 5-year colon cancer-specific-CID among age groups is statistically significant (p<0.001). The 5-year noncancer-specific CID were 2.2% (95%CI: 1.7–2.3%), 7.1% (95%CI: 7.0–7.5%), and 26.9% (95%CI:26.1–27.7%), respectively, and the difference of the 5-year colon noncancer-specific-CID among age groups is statistically significant (p<0.001). The 5-year colon cancer-specific CID was higher than noncancer-specific CID in patients <50 years of age, whereas the trend was reversed in patients >75 years of age. Noticeably, we found that the cumulative incidence of CCSM and NCSM in patients 50–75 years of age was similar during the 5-year follow-up period, as shown in Fig 2. In Figs 3–5, additional curves base on Kaplan-Meier method have been added to the cumulative incidence functions described in Fig 2.

### Subdistribution hazard models for cause-specific mortality

**Fig 6:**
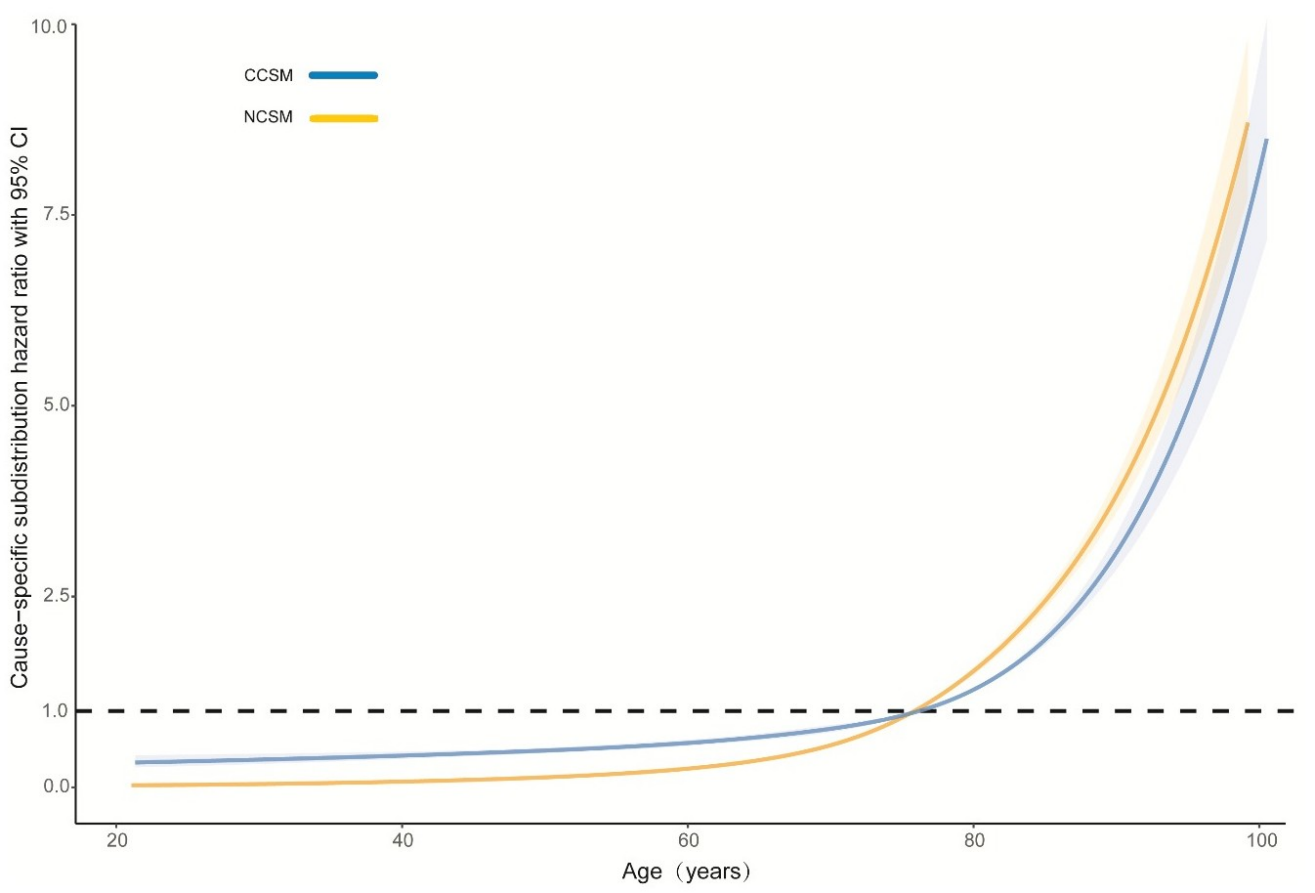
Age-varying association of cause-special mortality on age. CCSM: colon cancer-specific mortality; NCSM: noncancer-specific mortality.

In the next step, we performed multivariable analysis based on competing risk models for CCSM and NCSM, as shown in Table 2. Competing risk regression analysis demonstrated that age(per year increase, SHR 1.03,95% confidence interval [CI]: 1.03–1.04, per year) was an independent prognostic for CCSM. The effect values of some other covariates, including sex, race, marital status, tumor location, tumor size, histological type, histological grade and T stage on CCSM were statistically significant. On another hand, age was most predictive of NCSM: (SHR 1.08, 95% CI: 1.08–1.08, per year). As for NCSM, the effect values of sex, race, marital status, tumor location, histological type and T stage were statistically significant. The curve of cause-specific SHR with 95%CI for CCSM and NCSM according to patient age was shown in Fig 6. The SHR for CCSM or NCSM increased with increasing age. Further, we included patients with unknown information into the study, as shown in S1 Table.

**Table 2:** Multivariable competing risk analysis for CCSM and NCSM in stage I/II colon cancer.

Characteristic	CCSM			NCSM		
	SHR	95% CI	*P* value*	SHR	95% CI	*P* value*
**Age (per year)**	1.03	1.03–1.04	<0.001	1.08	1.08–1.08	<0.001
**Sex**						
Male	Ref.			Ref.		
Female	0.80	0.75–0.85	<0.001	0.68	0.65–0.72	<0.001
**Race**						
White	Ref.			Ref.		
Non-white	1.23	1.14–1.33	<0.001	0.87	0.81–0.93	<0.001
**Marital status**						
Married	Ref.			Ref.		
Unmarried	1.30	1.21–1.39	<0.001	1.31	1.24–1.38	<0.001
**Tumor location**						
Right-colon	Ref.			Ref.		
Left-colon	1.26	1.18–1.35	<0.001	0.94	0.89–1.00	0.04
**Tumor size, cm**						
<5	Ref.			Ref.		
≥5	1.11	1.04–1.19	0.002	0.99	0.94–1.04	0.58
**Histological type**						
Adenocarcinoma	Ref.			Ref.		
MC/SRCC	0.85	0.77–0.95	0.002	1.14	1.06–1.22	<0.001
**Histological grade**						
Well/Moderately	Ref.			Ref.		
Poorly/Undifferentiated	1.09	1.00–1.18	0.04	0.99	0.93–1.05	0.67
**T stage**						
T1-2	Ref.			Ref.		
T3	2.14	1.95–2.34	<0.001	0.91	0.87–0.96	0.001
T4	5.59	5.00–6.23	<0.001	0.72	0.65–0.80	<0.001

CCSM: colon cancer-specific mortality; NCSM: noncancer-specific mortality; SHR: subhazard ratio; CI: confidence interval; Ref.: reference; MC: mucinous carcinoma; SRCC; signet-ring cell carcinoma

* Based on competing risk analysis

### Cause-specific hazard models for cause-specific mortality

We also performed multivariable analysis based on cause-specific hazard models for CCSM and NCSM, as shown in Table 3. Multivariate analysis showed age was independently associated with CCSM (per year, hazard ratio, HR 1.04, 95% CI: 1.04–1.05) and NCSM (per year, HR 1.09, 95% CI: 1.09–1.10).

**Table 3:** Multivariable cause-specific hazard models for CCSM and NCSM in stage I/II colon cancer.

Characteristic	CCSM			NCSM		
	HR	95% CI	*P* value*	HR	95% CI	*P* value*
**Age, years**	1.04	1.04–1.05	<0.001	1.09	1.09–1.10	<0.001
**Sex**						
Male	Ref.			Ref.		
Female	0.75	0.70–0.80	<0.001	0.63	0.59–0.66	<0.001
**Race**						
White	Ref.			Ref.		
Non-white	1.21	1.12–1.31	<0.001	0.87	0.81–0.93	<0.001
**Marital status**						
Married	Ref.			Ref.		
Unmarried	1.36	1.27–1.45	<0.001	1.39	1.32–1.46	<0.001
**Tumor location**						
Right-colon	Ref.			Ref.		
Left-colon	1.27	1.19–1.36	<0.001	1.00	0.94–1.06	0.94
**Tumor size, cm**						
<5	Ref.			Ref.		
≥5	1.12	1.05–1.19	0.001	1.01	0.96–1.06	0.75
**Histological type**						
Adenocarcinoma	Ref.			Ref.		
MC/SRCC	0.86	0.77–0.95	0.002	1.11	1.03–1.19	0.01
**Histological grade**						
Well/Moderately	Ref.			Ref.		
Poorly/Undifferentiated	1.09	1.01–1.18	0.03	1.01	0.95–1.08	0.76
**T stage**			<0.001			0.45
T1-2	Ref.			Ref.		
T3	2.15	1.97–2.36	<0.001	0.98	0.93–1.04	0.47
T4	5.76	5.17–6.42	<0.001	1.04	0.94–1.15	0.50

CCSM: colon cancer-specific mortality; NCSM: noncancer-specific mortality; HR: hazard ratio; CI: confidence interval; Ref.: reference; MC: mucinous carcinoma; SRCC; signet-ring cell carcinoma

* Based on cause-specific hazard models

## Discussion

The present study evaluated colon cancer-specific and noncancer-specific CID by age-group and analyzed the impact of age on cause-specific mortality in patients with stage I/II colon cancer. We took competing events into consideration when evaluating survival outcomes and the results demonstrated that older patients had a poor colon cancer-specific survival and the higher incidence of noncancer-specific mortality. Further analysis indicated increasing age was a strong predictor of survival, with a more significant prognostic impact on NCSM.

Most previous studies used Kaplan-Meier and Cox proportional hazards models to investigate the association between age and the prognosis of colon cancer [5–8]. Both models consider that there is a single cause to the event of interest [10]. However, during long-term follow-up, there were more causes (so-called competing events) of deaths, especially for early stage cancers. Therefore, the competing risk should be considered in the survival analysis [11].

The strengths of our study lie in its comprehensive exploration of age-related CCSM and NCSM via competing risk analysis. In our cohort, we showed that CCSM increased with advanced age, which were in accordance with a previous population-based analysis demonstrating that older age was associated with decreased survival in early-stage disease [8]. Likewise, other studies have shown similar results [16, 17]. It has been reported that young patients presented poorer pathological features and advanced T stage compared with older patients, but were associated with a better OS and less CCSM [18, 19]. It may be explained that young patients are more likely to benefit from postoperative systemic treatment, because they are more able to tolerate toxicities associated with adjuvant therapy and suffer from fewer complications.

As age increases, the risk of competing events increases in cancer patients, which will increase the complexity of cancer treatment and affect patient survival [20–23]. It is important to take competing events into consideration when evaluating the prognosis in cancer patients. However, few studies focused on NCSM in colon cancer. Herein, we used competing risk model and the results indicated older patients were at a higher risk of NCSM. The results were in line with a retrospective study including a cohort of 19415 patients showing that age was associated with increased probabilities of competing mortality [24]. One of the possible explanations might be that age-related comorbidities result in a higher risk of NCSM in older patients.

Noticeably, we found a difference between CCSM and NCSM among age-group (*P*<0.001). Patients <50 years of age were associated with more invasive features but fewer CCSM, while older patients had a significantly higher risk of CCSM and NCSM. Particularly, 5-years NCSM was significantly higher than 5-years CCSM (26.9% vs 16.1%) in patients >75 years of age, which emphasized the importance of focusing on competitive events in survival analysis. A possible explanation was that older patients usually received less treatment and may not benefit from active treatment. Given that patients >75 years of age are often excluded from clinical trials, the benefits of surgery for older patients should be carefully balanced with the patient’s life expectancy. Effective perioperative management and better basic life support are crucial parts for elderly patients to improve prognosis. On the other hand, more aggressive treatment should be given priority to younger patients because CCSM is the leading cause of death. Interestingly, in patients 50–75 years of age, we found a similar cumulative incidence of CCSM and NCCSM. Therefore, we further used multivariable competing risk analysis to further assess the impact of age on prognosis, and the results demonstrated that age was a strong predictor of survival, with a more significant prognostic impact on NCSM. The curve of cause-specific SHR according to patient age was shown the SHR for CCSM or NCSM increased with increasing age. Our results showed the cumulative incidence of CCSM and NCSM were overestimated when using the Kaplan-Meier method, as shown in Figs 3 to 5. The results are similar to some previous researches [9, 11]. Thus, in the presence of a high proportion of competing events, it is important to take competing events into consider when estimating the risk of colon cancer mortality. In our study, the outcome “cause-specific death” was identified used SEER cause-specific death classification. Some previous studies including breast cancer and prostate cancer also used SEER database to identify cause-specific death and performed competing risk analysis [25, 26].

There are several potential limitations. First, there is the likelihood of miscoding. However, miscoding would likely be random, which unlikely to introduce systematic misclassification bias. Second, the SEER database lacks several tumors- and treatment-related information (e.g. perineural infiltration, postoperative adjuvant therapy, and quality of surgery), and thus our analysis could not adjust for these potential confounders. Adjuvant therapy is essential to survival outcome. However, our study cohort included patients with stage I/II colon cancer, and whether patients with stage II colon cancer can benefit from adjuvant therapy remains controversial. Third, comorbidity, frailty and functional status were not recorded in the SEER database, and could not be evaluated in our study. Age is closely related to comorbidities and frailty. And it can offset the limitation in some degree. Despite these limitations, our study was a population-based analysis from the SEER database, making the results convincing to the clinicians. Additionally, this study provides information on the impact of age on cause-specific mortality in age groups and might be helpful in treatment decision making.

## Conclusion

In this study, we indicated that age is a predictor of colon cancer-specific and noncancer-specific mortality in patients with stage colon I/II cancer. Older patients were more likely to suffer from noncancer-specific mortality, the benefits of surgery for older patients should be carefully balanced with the patient’s life expectancy.

## Supporting information

S1 Table Multivariable competing risk analysis for CCSM and NCSM in stage I/II colon cancer included patients with unknown demographic and clinicopathological data. CCSM: colon cancer-specific mortality; HR: hazard ratio; CI: confidence interval; Ref.: reference; MC: mucinous carcinoma; SRCC; signet-ring cell carcinoma. * Based on competing risk analysis.
(DOC)

S1 Dataset (XLS)
