# Supplementary material for: Association of age and cause-special mortality in patients with stage I/ II colon cancer: A population-based competing risk analysis
Source: PLoS One. 2020 Oct 16;15(10):e0240715. doi: 10.1371/journal.pone.0240715 (PMC7567365; doi:10.1371/journal.pone.0240715)
Supplement: S1 Table — CCSM: colon cancer-specific mortality; HR: hazard ratio; CI: confidence interval; Ref.: reference; MC: mucinous carcinoma; SRCC; signet-ring cell carcinoma. * Based on competing risk analysis. (DOC) [file pone.0240715.s001.doc]

S1 Table. Multivariable competing risk analysis for CCSM and NSCM in stage I/II colon cancer included patients with unknown demographic and clinicopathological data

| **Characteristic** | **CCSM** | | | **NCSM** | | |
| --- | --- | --- | --- | --- | --- | --- |
|  | **SHR** | **95% CI** | ***P* value*** | **SHR** | **95% CI** | ***P* value*** |
| **Age, years** | 1.03 | 1.03-1.04 | <0.001 | 1.08 | 1.08-1.08 | <0.001 |
| **Sex** |  |  |  |  |  |  |
| Male | Ref. |  |  | Ref. |  |  |
| Female | 0.82 | 0.77-0.87 | <0.001 | 0.67 | 0.64-0.71 | <0.001 |
| **Race** |  |  |  |  |  |  |
| White | Ref. |  |  | Ref. |  |  |
| Non-white | 1.21 | 1.12-1.30 | <0.001 | 0.87 | 0.82-0.93 | <0.001 |
| Unknown | 0.28 | 0.09-0.86 | 0.03 | 0.19 | 0.06-0.60 | 0.005 |
| **Marital status** |  |  |  |  |  |  |
| Married | Ref. |  |  | Ref. |  |  |
| Unmarried | 1.28 | 1.20-1.36 | <0.001 | 1.32 | 1.26-1.39 | <0.001 |
| Unknown | 1.18 | 1.02-1.36 | 0.03 | 1.09 | 0.97-1.22 | 0.13 |
| **Tumor location** |  |  |  |  |  |  |
| Right colon | Ref. |  |  | Ref. |  |  |
| Left colon | 1.27 | 1.19-1.36 | <0.001 | 0.93 | 0.88-0.98 | 0.004 |
| **Tumor size, cm** |  |  |  |  |  |  |
| <5 | Ref. |  |  | Ref. |  |  |
| ≥5 | 1.11 | 1.04-1.18 | 0.002 | 0.98 | 0.93-1.03 | 0.39 |
| Unknown | 0.92 | 0.79-1.08 | 0.27 | 1.02 | 0.93-1.12 | 0.71 |
| **Histological type** |  |  |  |  |  |  |
| Adenocarcinoma | Ref. |  |  | Ref. |  |  |
| MC/SRCC | 0.89 | 0.81-0.98 | 0.02 | 1.12 | 1.05-1.20 | <0.001 |
| **Histological grade** |  |  |  |  |  |  |
| Well/Moderately | Ref. |  |  | Ref. |  |  |
| Poorly/Undifferentiated | 1.09 | 1.01-1.18 | 0.03 | 0.99 | 0.93-1.06 | 0.85 |
| Unknown | 1.16 | 0.95-1.40 | 0.14 | 0.90 | 0.78-1.04 | 0.15 |
| **T stage** |  |  |  |  |  |  |
| T1-2 | Ref. |  |  | Ref. |  |  |
| T3 | 2.28 | 2.09-2.48 | <0.001 | 0.93 | 0.88-0.98 | 0.003 |
| T4 | 5.89 | 5.31-6.54 | <0.001 | 0.74 | 0.67-0.82 | <0.001 |

CCSM: colon cancer-specific mortality; NCSM: noncancer-specific mortality; SHR: subhazard ratio; CI: confidence interval; Ref.: reference; MC: mucinous carcinoma; SRCC; signet-ring cell carcinoma

* Based on competing risk analysis
